# Automated Classification of Glandular Tissue by Statistical Proximity Sampling

**DOI:** 10.1155/2015/943104

**Published:** 2015-01-18

**Authors:** Jimmy C. Azar, Martin Simonsson, Ewert Bengtsson, Anders Hast

**Affiliations:** Centre for Image Analysis, Department of Information Technology, Uppsala University, 75105 Uppsala, Sweden

## Abstract

Due to the complexity of biological tissue and variations in staining procedures, features that are based on the explicit extraction of properties from subglandular structures in tissue images may have difficulty generalizing well over an unrestricted set of images and staining variations. We circumvent this problem by an implicit representation that is both robust and highly descriptive, especially when combined with a multiple instance learning approach to image classification. The new feature method is able to describe tissue architecture based on glandular structure. It is based on statistically representing the relative distribution of tissue components around lumen regions, while preserving spatial and quantitative information, as a basis for diagnosing and analyzing different areas within an image. We demonstrate the efficacy of the method in extracting discriminative features for obtaining high classification rates for tubular formation in both healthy and cancerous tissue, which is an important component in Gleason and tubule-based Elston grading. The proposed method may be used for glandular classification, also in other tissue types, in addition to general applicability as a region-based feature descriptor in image analysis where the image represents a bag with a certain label (or grade) and the region-based feature vectors represent instances.

## 1. Introduction

There have been many attempts over the past decades for automating cancer grading in tissue, most notably in breast and prostate tissue, where the standard scoring systems in use are the Elston [1] and Gleason [2] grading systems, respectively. The first computerized grading of prostate tissue was published in 1978 [3]. More recently, high classification rates were obtained for the simple case of discriminating between low-grade and high-grade cancer in prostate tissue [4–7]. There have been attempts in [4, 5] at performing the classification task and extracting a large and diverse feature set including color, texture, morphometric, fractal, and wavelet features. Often this is followed by a feature reduction method such as sequential forward feature selection as in [4] or similar greedy algorithms, which, though being suboptimal approaches, are motivated by the fact that the feature set is large and a brute force or branch-and-bound method may become intractable or computationally inefficient.

Often a main factor that limits automated classification lies not in the choice of classifier but in the choice of feature set. The discriminative ability of a classifier is limited by the extent to which the classes themselves are separate in feature space. For well-represented classes, the intrinsic overlap and proximity of the classes in feature space determine the upper limit on the classification rate. The chosen features define the extent to which the classes overlap. The selection of a large number of different types of features is common in practice and is often an indication of lack of knowledge as to what features exactly have discriminative power; instead it reflects speculation over which features may prove useful or may have a contributing role [8]. However, choosing a plethora of features, whether informative or not, increases the dimensionality of the feature space and often exposes the classification task to the peaking phenomenon [9]. Furthermore, this shifts the burden of the problem toward feature selection or extraction which is often difficult to solve in a manner that is true to the final objective (i.e., the final classification rate), and this is due to computational limitations and the prevalence of either suboptimal criteria or criteria that are often not aligned with the final objective. It is therefore important to select a discriminative set of features that is able to separate among the different classes.

Automated tissue grading is very difficult for several reasons. One reason lies in the difficulty of translating the experience and observations of the human expert, that is, the trained pathologist, into well-defined features that can be extracted automatically from the image. Moreover, due to the complexity of the tissue structure and subjectivity of the grading process, especially among the intermediate grades, there is no clear consensus as to which features or combination of features is to be used consistently. Upon deeper examination, we find that experts' rules tend to eventually branch out into increasingly complicated conditions and exceptions. This leads to countless if-else situations where exceptions eventually outgrow the norm. There is therefore a problem in identifying features explicitly, and moreover even when such features have been suggested by pathologists, the complexity and variability of the images and tissue structures in addition to variables relating to stain absorption can still obstruct the extraction of such features in a reliable manner that allows for automation.

In general, there is a sensitive balance between overadapting to the complexity of the problem on the one hand and weakly accounting for it on the other hand such as the case when extracting global texture features without taking into account any knowledge of tissue architecture. Both of these extreme approaches may lead to inadequate results and an inability to generalize well. In the approach that we propose, we avoid the explicit extraction of structure properties (such as nuclei shape, glandular unit shape, and thickness of epithelium layer) beyond a rough decomposition of images into a few classes based on the staining. Yet, the method is still strongly founded on the architecture of glandular tissue (such as breast or prostate) and relies upon detecting glandular lumen and tissue components as a starting point. We use sequential region expansion to sample the space around lumen regions in the form of rings and preserve the statistics and component ratios within these rings in order to describe and represent these regions in an implicit manner. During the progression of cancer into advanced stages, when a glandular unit transforms into cribriform shape or splits into multiple lumen regions, such a phenomenon should be detected by the method due to the unusual presence of lumen and other structures in the outer sampling rings which reflects on the shape of the extracted profile curve and consequently on its classification and labeling.

As opposed to most local neighborhood sampling or* bag of features* methods that either are patch-based or result in orderless, histogram-based features [10, 11], the method we propose does the sampling around a given (lumen) region as opposed to a pixel, while preserving the region's boundary shape and encoding spatial distance from it. The contributions of our work can be stated as follows.

1. We present a new approach to encode features in complex tissue images such as prostate and breast. The approach called* statistical proximity sampling* relies on a method of boundary expansion around lumen regions; it uses rings or neighborhood strips around these regions while preserving the boundary shapes.
2. The method is able to simultaneously encode the relative quantitative proportions of each tissue type around a lumen region as well as the spatial distribution of these proportions from the central lumen region, resulting in highly descriptive and discriminative features.
3. Combining this neighborhood-based feature description with multiple-instance learning, we are able to represent complex images in an efficient and information-preserving manner, which is more consequential than representing an entire image with a single feature vector.

To highlight the context of our work, we briefly describe below the Elston and Gleason grading systems and how our method relates to some important aspects of these.

The Elston score is based on three different components. The first is tubular formation or “tubularity,” where the presence of glandular tissue in the sample is given a score from 1 to 3, ranging, respectively, from healthy tissue (prevalently glandular) to solid tumors (scarcely glandular). The second component is nuclear pleomorphism and is concerned with nuclear size, shape, and chromatin texture; this attribute is also assigned a score from 1 to 3 depending on the morphological irregularities of nuclei. The third component of grading is mitotic activity which corresponds to growth rate and is determined by counting dividing cells, ranging from a low cell count (score 1) to a high cell count (score 3). The final, high-level Elston grade is then derived by summing up the individual scores from the three parts: a sum of 3–5 points is defined as Elston grade I, 6-7 as grade II, and 8-9 as grade III.

Analogously, Gleason grading for prostate is based on five patterns, which are highly dependent on tissue architecture and the description of glandular units. The patterns from 1 to 5 are described by how glands alter form while transitioning from small, well-defined, and closely packed units, corresponding to well-differentiated carcinoma (pattern 1), to larger glandular units with increased interglandular distances, corresponding to moderately differentiated carcinoma (pattern 2), until the glands are no longer recognizable and cells start to invade surrounding tissue in neoplastic clumps. In pattern 5, the tissue does not have any, or only a few, recognizable glands.

Thus, in conclusion, both Gleason grading and the first component of Elston grading are based on patterns that are defined by the amount and architecture of glandular units and tubules present in the tissue sample. The ability to identify tubules and glandular structures is an essential requirement for both grading systems. While there is a lot of work on identifying nuclear pleomorphism and mitotic count, as most recently in [12], our contribution in this paper is to propose a new effective way of extracting information concerning glandular architecture, which is directly related to the first component in Elston grading and is an essential part of Gleason grading. In particular, what we present in this paper is a method that enables us to distinguish between images with tubular structures, denoted by *C*1, and images lacking tubular structures, denoted by *C*0, where these images are taken from both healthy and cancerous breast tissue, since we want to be able to identify tubules in both healthy and cancer tissue samples.

## 2. Materials and Methods

### 2.1. Statistical Proximity Sampling

The grading of cancerous tissue of glandular organs such as prostate and breast is to a large extent based on the tissue architecture around the glandular lumen regions. In previous work [13, 14], we have presented automated methods for color decomposition and pattern-based image segmentation that result in density or probability maps, one per stained tissue type. In the current work, we present a method that uses such types of maps as input for deriving a set of features based on statistically sampling the neighborhood of lumen regions. The purpose is to discriminate between tubule and nontubule regions in breast tissue sections.

Our method proceeds in the following manner.

**Figure 1:**
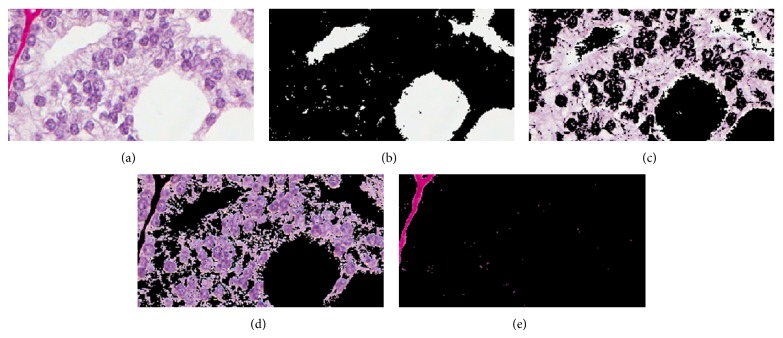
An image of prostate tissue (a) is decomposed into four classes: lumen, epithelium, nuclei, and stromal regions. The probability maps in this example were thresholded at a level of 10% for enhancing visibility.

**Figure 2:**
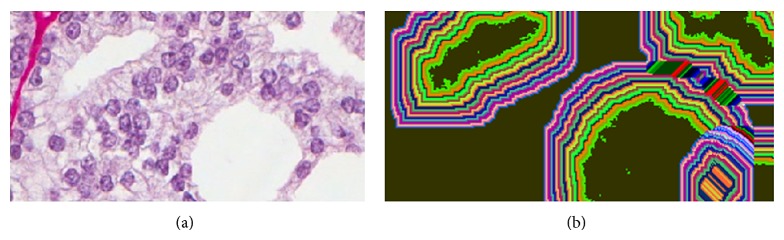
The sampling rings growing away from each lumen region for the example in Figure 1. Each ring is obtained by first dilating the lumen region and then subtracting it from the dilated version. This is done sequentially and the number of rings in this example is 30.

1. A tissue image is softly classified into a set of *K* probability maps using any method such as color decomposition (see [14]) or a pattern analysis approach (see [13]); an example is shown in Figure 1, where the *K* tissue types correspond to lumen, epithelium, nuclei, and stroma.
2. Starting from the lumen regions, each region is separately dilated by a square structuring element in sequential unit steps forming a set of rings or annuli around the original lumen space (see Figure 2). These rings are regarded as neighborhood strips from which we will gather statistics on the quantity and location of surrounding tissue types. The boundary shape is preserved within a reasonable number of sampling rings.
3. Within each ring, we compute the proportions of the different tissue types (lumen, epithelium, nuclei, and stroma) using the derived probability maps. Thus, for each ring, we obtain a vector of length *K*. For instance, a vector such as [0.2, 0.4, 0.1, 0.3] in a given ring indicates that the relative proportions of lumen, epithelium, nuclei, and stroma are 20%, 40%, 10%, and 30%, respectively.
4. The vectors obtained in step (3) are stacked, forming a single vector of length *R* × *K*, where *R* is the number of rings used, that is, neighborhood strips. Thus each lumen region from step (1) will be represented by such a feature vector of length *R* × *K*. An example of these vectors is shown in Figure 4, where there are 4 lumen regions and consequently 4 such feature vectors plotted using different colors.
5. We present each image as a bag or collection of feature vectors corresponding to lumen regions in the image. Thus, we use multiple-instance learning to represent an image using a collection of feature vectors and perform the classification of each image based on its contents. We also use the bag dissimilarity approach [15] to decouple the classification task from the multiple-instance formulation, allowing us to use any type of classifier without difficulty.

In step (2), it is possible to use dilations with larger steps or with a larger structuring element when deriving the rings. This would make the collected statistics less noisy but would also decrease the spatial resolution of the collected data (analogous to the effect of applying a moving average filter).

The method is designed to be applicable to any glandular tissue type. We have developed and tested it on breast and prostate tissue. We begin by explaining the method through an example for the case of prostate tissue. Figure 1 shows a cross-section of prostate tissue that has been stained with a Sirius-hematoxylin stain combination along with the resulting image decomposition into four probability maps which represent in this case the classes corresponding to lumen, epithelium, nuclei, and stromal regions. Note that the decomposition method used for prostate tissue follows from our previous work in [14]. The proximity sampling method takes as input the probability maps generated from the decomposition, regardless of which method was employed for the latter. The image selected for decomposition in Figure 1 is a cropped image of size 183 × 339 and was chosen to contain only a small number of lumen regions so that the number of feature profiles that follow remains tractable for display. The probability maps were automatically rearranged according to a descending order of mean intensity value. This allows the automatic selection of the lumen class as the first image in this ordered sequence.

In what follows, we discuss in detail how the main feature vector of the statistical proximity sampling method is obtained. The method proceeds by statistically sampling the neighborhood around each lumen in terms of class component quantities. By sequential dilation of the lumen region and subtraction of the preceding area, we obtain concentric rings or annuli progressing spatially away from the lumen in either inward, outward, or both directions, extending the lumen shape (see Figure 2). Within each ring, the fraction of each class component, that is, lumen, epithelium, nuclei, and stroma, is computed as a ratio of the sum of class posterior probabilities within the ring to the total area of the ring. These are then concatenated into a vector with *K* = 4 parts, where *K* is the number of classes. The number of dilations or rings we have used in this case for illustration was 30. This creates a profile of how these class quantities are changing spatially as one moves away from the lumen within its neighborhood. As cancer progresses from benign to malignant, the different grades of cancer are expected to exhibit different patterns in terms of the quantities and order of these class components around the lumen which would result in different profile curves sampled from these rings. The features represent fractional values in the range [0,1]. The shape of the curve captures spatial (order) information and represents statistical quantification of the classes.

**Figure 3:**
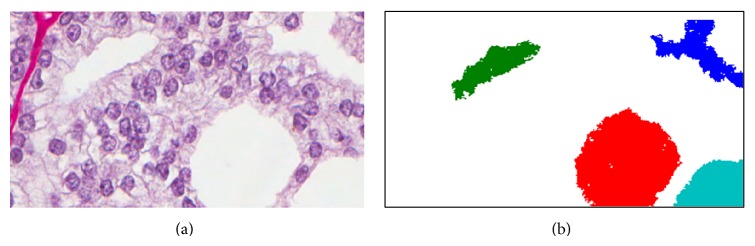
The lumen class from the original image shown in Figure 1 is extracted. The different regions are labeled according to their 4-connectivity. These regions form the basis and starting point of our algorithm for deriving features.

**Figure 4:**
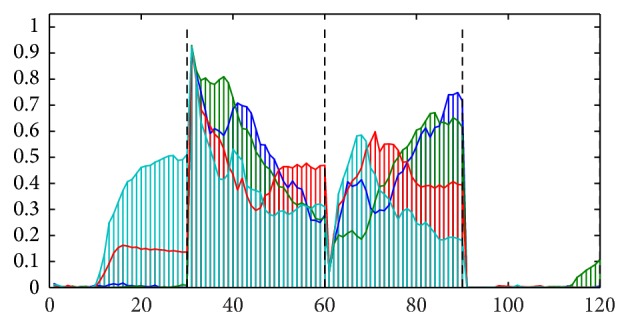
Feature vectors are shown, one for each of the four lumen regions illustrated in Figure 3. The vectors are divided into 4 parts delineated by black vertical lines: the first part depicts the first 30 elements representing the fraction of lumen within the 30 sampling rings, the second part depicts those for the epithelium component, the third part depicts those for the nuclei component, and the fourth part depicts those for the stromal component.

Figure 3 shows lumen regions extracted from the corresponding posterior map and labeled according to their 4-connected neighborhood; that is, pixels that are adjacent diagonally are not considered neighbors. Figure 4 shows the profile curves obtained, one for each of the four lumen regions. The first 30 elements represent the changing amount of neighboring lumen within those rings; the second part consisting of another 30 elements represents that of epithelium, the third part that of nuclei, and the fourth that of stroma. To validate and understand what the curves represent, one should compare the profile of each lumen to its spatial neighborhood shown in Figure 3. The colors shown in Figure 4 have been set to match those lumen regions shown in Figure 3. For example, the cyan curve represents the cyan colored lumen region. From Figure 3, we notice that the sampling rings should contain a considerable fraction of lumen due to the large neighboring lumen region shown in red color. Consequently, the first part of the cyan curve shows high values. Similar analysis follows for the other parts of the curve in which one can see how each class component varies as one moves away from the lumen region. The fourth part of the curve is particularly easy to notice since there is no stromal component close enough to the cyan luminal region.

**Figure 5:**
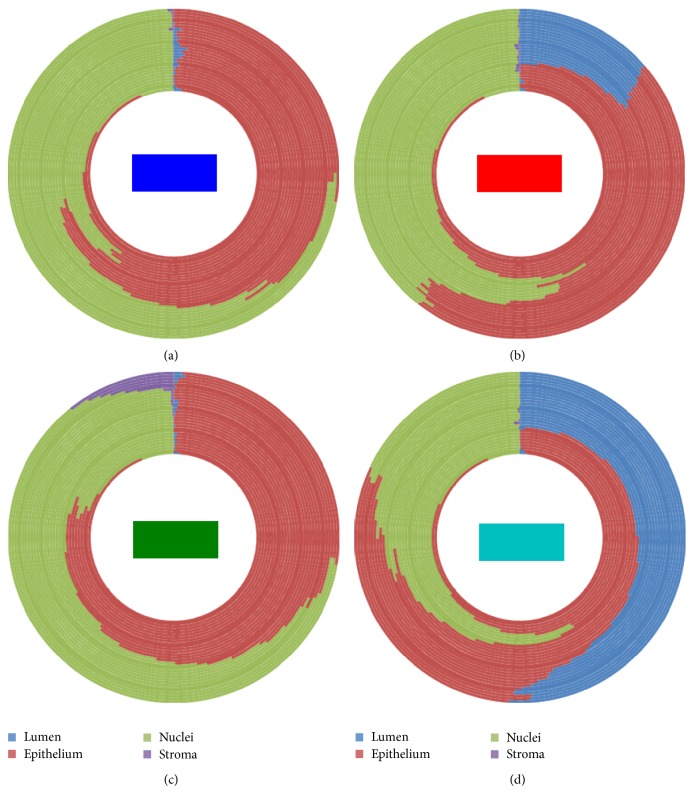
The four parts of each feature vector illustrated in Figure 4 are depicted in a relative frequency graph for each luminal region in this example. The color of the central rectangle in each subplot indicates the corresponding lumen region shown in Figure 3.

Alternatively, in order to show how the different luminal regions compare to each other in terms of the spatial composition of their proximities, we replot the curves of Figure 4 such that the proportions of the four different tissue types (lumen, epithelium, nuclei, and stroma) across the rings are shown in a relative frequency pie chart for each luminal region separately. This is illustrated in Figure 5, where each subplot represents a luminal region indicated by the color of the central rectangle, and we note here that each of these exhibits a different profile.

**Figure 6:**
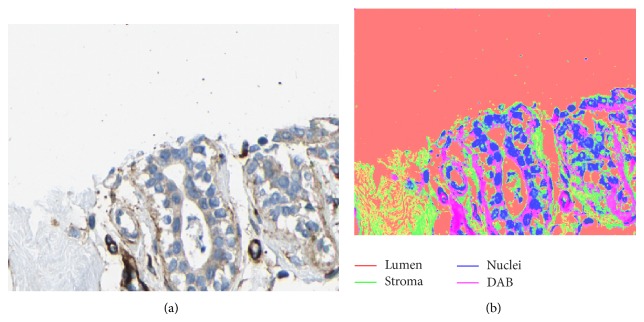
An image of breast tissue and its resulting decomposition into four classes: lumen, stroma, nuclei, and DAB regions.

**Figure 7:**
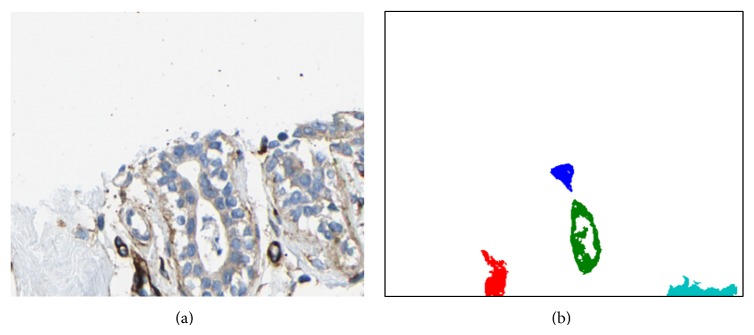
A few selected regions from the lumen class of the original image shown in Figure 6 are extracted. The different regions are labeled according to their 4-connectivity and shown using different colors.

**Figure 8:**
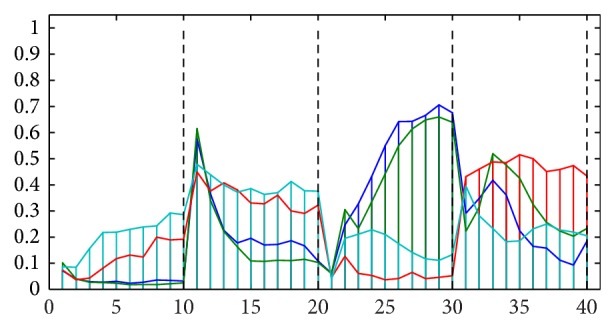
A feature vector is shown, one for each of the lumen regions illustrated in Figure 7. The vectors are divided into 4 parts delineated by black vertical lines: the first part depicts the first 10 elements representing the fraction of lumen within the 10 sampling rings, the second part depicts those for the stroma component, the third part depicts those for the nuclei component, and the fourth part depicts those for the DAB component.

We note that the previous example was based on the spectral decomposition of tissue that was specifically stained (using Sirius-hematoxylin) in order to express the different relevant tissue components (see [14]). However, in order to illustrate that the proposed concept is robust and generally applicable, we have also applied it to images of breast cancer tissues from the Human Protein Atlas database [16] which are stained using hematoxylin-eosin + DAB to visualize general background tissue structures and specific proteins. As an example, we show a cropped image region of size 362 × 450 selected from the case “Group *R*”—*C*1 in [13].  Figure 6 shows the decomposition of the image into four classes corresponding to lumen, stroma, nuclei, and DAB.  Figure 7 shows four selected lumen regions from the posterior map of the lumen class in order to display their respective feature curves as based on the proximity sampling method described above. Finally, Figure 8 shows the profile curves for this example, where the number of sampling rings around each lumen was set to 10. In a similar manner to the previous example, several detailed conclusions may be drawn from these figures; however the most general one is that these feature curves capture the statistical distribution of the classes around each lumen region and may therefore be used to classify those regions.

### 2.2. Bag Dissimilarity for Classification

**Figure 9:**
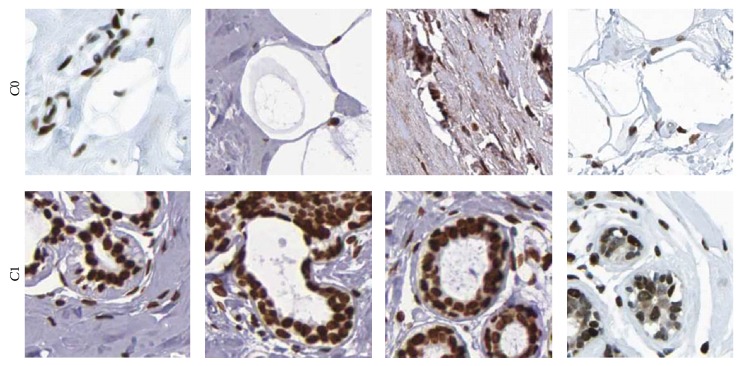
A few sample cases of the breast dataset consisting of images of breast tissue sections labeled as *C*1 and *C*0 based on the presence or absence of milk ducts, respectively.

To test our method, we used a dataset consisting of images of breast tissue sections obtained from the Human Protein Atlas project [16], where every image has been assigned a malignancy grade by an expert. The assigned class labels denoted by *C*0 and *C*1 are associated with the tubule-based Elston grading, where the main factor is the absence (*C*0) or presence (*C*1) of milk ducts in the tissue. Sample images of the dataset are shown in Figure 9. Note that all microscopy images of the given dataset were acquired under the same magnification level of ×40. The dataset consists of tissue sections containing cells, glands, and luminal regions, and the proximity sampling method we have proposed applies in general also for images of tissue types that contain similar structures in living organisms.

In the dataset, an image may contain several lumen regions. A feature vector is derived for each of these lumen regions using the proximity sampling method described. The aim is to train a classifier on the labeled, that is, graded, images in order to predict the label of a new image automatically. Thus, there is an inherent relation between the formulation of this problem and multiple-instance learning [17]. In the context of the latter, the feature vectors derived from the lumen regions in an image may be regarded as “instances” or objects and the image itself as a “bag” or compound object consisting of one or more instances. The instances themselves are not labeled, but rather only the bag carries a label, which in this case is the tubule-based grade assigned by the pathologists. Also different bags may contain a different number of instances. Some of the instances in a bag may be less important in contributing to the bag label, whereas one or more may be key instances, belonging to the so-called “concept,” that significantly define the bag label. For example, an image may contain one gland unit that characterizes a grade 3 cancer region, in addition to several noncontributing, background lumen regions. In such a case, one of the instances belongs to the “concept” that contributes to the grade 3 label.

The multiple-instance learning approach is more flexible than standard classification approaches in that the representation allows us to encode more information from a single image by considering it as a collection of feature vectors rather than encoding the entire image by a single feature vector. Images of real life objects (such as tissue sections) often contain a lot of important subregions with different characteristics and may be therefore too complex to be represented by a single feature vector [15]. The multiple-instance representation is highly informative in this situation since it encodes information from different regions in an image, each of which may contribute to the final grade or label of the image as a whole.

However, the classification task that ensues becomes more complex as a classifier is trained and optimized over the dataset. Therefore, in order not to add complexity to the construction of a classifier and preserve the flexibility of the task, we follow the bag dissimilarity approach described in [15], which does not attempt to locate a “concept” but rather uses a similarity measure across bags, which are seen as sets of instances. The dissimilarities computed between the bags become the new features, and this allows us to construct any classifier in this new feature space, thus decoupling the original multiple-instance problem from the classification task itself. Moreover, the bag dissimilarity approach allows us to consider multiclass data, that is, data with several grades, whereas in the traditional multiple-instance learning problem only two classes, namely, a positive and a negative class, are considered at any given time, and a one-against-one or one-against-all approach is often used in the classification of multiclass situations.

### 2.3. Additional Lumen Shape Features

Insofar, we have presented a new vectorial proximity-based feature for describing tissue architecture around glands, and we proceed in the next section to demonstrate its usefulness as a feature descriptor. However, in order to evaluate whether more conventional scalar features add any information to the new feature, we have implemented four classical, well-known scalar features that are simple to compute from each lumen region. The first is the size of the region, while the other measures relate to its shape and are the bending energy, area-to-perimeter ratio, and convexity ratio. Bending energy [18] is defined around the lumen perimeter based on the chain code sequence and is given by *E*_*b*_ = ∑_*p*=1_^*P*_*f*_^ *κ* ^2^(*p*), where *κ*(*p*) is a smoothed version of the curvature signal *θ*(*p*) = tan^−1^⁡((*y*_*c*_(*p*) − *y*_*c*_(*p* − 1))/(*x*_*c*_(*p*) − *x*_*c*_(*p* − 1))), where (*x*_*c*_(*p* − 1), *y*_*c*_(*p* − 1)) and (*x*_*c*_(*p*), *y*_*c*_(*p*)) are two consecutive points of the curvature. The minimum value is 2*π*/*R* and is attained for a circle of radius *R*. This feature is an indicator of convexity/concavity of the lumen boundary. The area-to-perimeter ratio is defined as (4*πA*)/*P* ^2^, where *P* is the perimeter and *A* the area of the lumen region. Convexity is defined as *A*_lumen_/*A*_convex  hull_, where *A*_lumen_ is the area of the lumen region and *A*_convex  hull_ is the area covered by the convex hull encompassing the lumen region. This ratio is in the range [0,1] and is closer to 1 when the lumen shape is convex and closer to 0 when highly irregular such as the case of cribriform grade 3-4 glandular units in prostate tissue, for instance. These lumen shape features are then compared with the main proximity feature vector, and classification results are presented in Section 3.

## 3. Results and Discussion

For each image in the dataset, we have used our proximity-based feature method to obtain a set of descriptive features. Then we used multiple-instance learning to represent each image as a bag of instances and transform the feature space into a dissimilarity space by computing the distances among the different bags. The MIL toolbox and various classifiers were used for this purpose [19, 20]. Our dissimilarity matrix is computed among the bags based on the linear assignment distance measured between sets [15, 21]. The dataset is then randomly split into a training set and a test set, and cross-validation procedures are used throughout. The mean error and standard deviation are then reported for both datasets. The characteristics of our dataset are summarized in Table 1. Note that ten sampling rings were used for the statistical proximity sampling method throughout all cases resulting in 40-element feature vectors for the case of the breast dataset since the number of classes was four using the hematoxylin-eosin + DAB stain.

**Figure 10:**
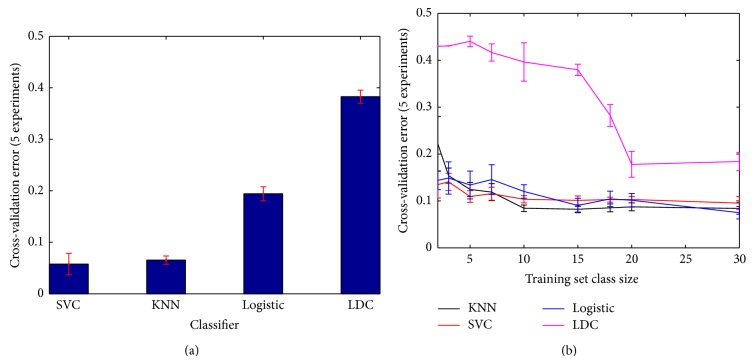
Classification results for the breast dataset, using only the features derived by statistical proximity sampling. Classifiers used are the linear support vector classifier (SVC), *k*-nearest neighbor classifier (KNN), the logistic classifier (Logistic), and normal-based linear discriminant classifier (LDC). (a) 10-fold cross-validation error. (b) Classifier learning curves. Error bars represent one standard deviation.

Figure 10 shows the classification rates and classifier learning curves using only the features derived by the statistical proximity sampling method. Note that the parameters for the support vector classifier and *k*-nearest neighbor classifier were optimized using leave-one-out cross-validation over a training set comprising randomly 25% of the original dataset. The 10-fold cross-validation rates for all classifiers over the remaining test set are then computed. The entire process is further repeated in 5 experiments. The results are presented in Table 2. The highest classification rates were obtained using the *k*-nearest neighbor classifier and the support vector classifier with 93.4% and 94.2% correct classification for the breast dataset, respectively. Note that assigning misclassification costs for different classes may be set as desired through the regularization parameter of SVC if needed. For comparison, a similar procedure was applied, however, using only the 4 classical lumen shape features that were described in Section 2.3. The classification rates obtained were much lower with the best performance at 62.69%.

### 3.1. Unsupervised Approach

**Figure 11:**
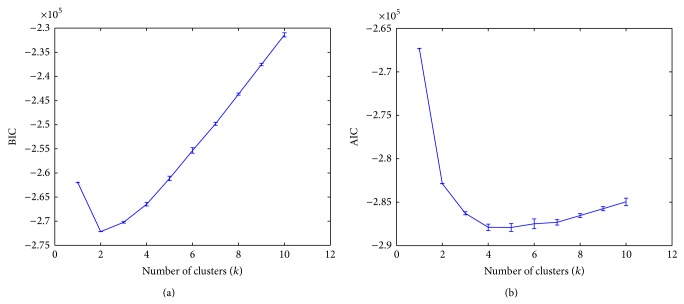
Optimal number of clusters using the Bayesian information criterion (BIC) and Akaike information criterion (AIC) over the breast dataset. The error bars represent the standard deviation at each value of “*k*.”

Although we do not explore unsupervised methods for malignancy grading in this paper, we would like to highlight the possibility of applying a clustering-based approach coupled with an information criterion for classifying images. We demonstrate in what follows how clustering may be applied to classify instances in the absence of bag labels. In other words, we attempt to identify and locate key clusters or groups of instances forming main clusters. A bag label (i.e., image grade) may then be obtained using a voting scheme over the cluster labels of the instances that belong to it. To study whether there may be an inherent number of clusters in the data possibly due to a certain fixed number of neighborhood descriptions that tend to recur around lumen regions, we used the Bayesian information criterion (BIC). We clustered the breast dataset using the Gaussian mixture model (GMM) several times with a varying number of clusters *k* ranging from 1 to 10, and we computed the BIC values as defined by BIC = −2ln⁡⁡(*L*) + *k*ln⁡⁡(*n*), where *n* is the number of objects in the data, *L* is the likelihood value of the mixture fit, and *k* is the number of clusters. Note that the mixture model was initialized randomly 10 times for each value of *k*. As the number of clusters increases, we expect the log-likelihood to increase monotonically; however the BIC measure also includes the model parameters into the tradeoff, which in this case is the number of clusters *k*. The optimal mixture model would have a high log-likelihood yet at a lowest possible complexity *k*. A plot of the BIC values versus the number of clusters is shown in Figure 11. We deduce in this case that for the breast dataset the optimal number of clusters at which the BIC curve attains a minimum is 2. This result does not necessarily imply that there are 2 clusters in the data in an absolute sense. When using the Akaike information criterion (AIC), we note in Figure 11 that the optimal number of clusters at which the curve attains a minimum becomes 4, since AIC in this case penalizes model complexity less heavily than BIC and thus results in the selection of a larger model. Conclusively, this might suggest that a model selection of 2, 3, or 4 classes in this case is a reasonable choice.

## 4. Conclusions

We have presented a general and simple method for statistically describing the distribution of glandular structures around lumen regions. The method makes use of sampling based on an iterative region expansion procedure that preserves the shape of the lumen areas. One advantage of this approach is that, by analyzing the neighborhoods of lumen regions and preserving the spatial and statistical information in these proximities, we avoid the need to extract explicit features concerning the underlying tissue structures themselves. The result is a set of feature vectors containing spatial and statistical information that may be used to describe regions in tissue images for a large variety of purposes, among which is tubule-based grading, as we have demonstrated in this paper. The input required for the method can be either a set of probability or binary maps derived from soft or crisp classification regardless of the supervised or unsupervised method (e.g., [13, 14]) used to generate these maps. The method is also robust and its dependence on the quality of these maps is minimal since the approach does not attempt to derive any precise cellular or subcellular features, which would require accurate image segmentation.

Due to the natural complexity of biological tissue and the grading process, we have avoided the single feature vector based representation used in standard pattern recognition. Automated grading was instead done using a bag dissimilarity approach while treating the problem in a similar manner to multiple-instance learning. Since images of tissue sections often contain various spatial subregions which may have completely different properties and characteristics, such an approach is more capable of encoding the diverse content and level of information represented in these images. Classification results using cross-validation have shown that the statistical proximity sampling method presented is able to provide a set of discriminative features for tubule-based cancer grading.

A possible drawback of the dissimilarity approach we have used in our classification is that although the classification task itself is accomplished and the diagnosis is automated, no single “concept” is identified during the process, as it remains hidden. However, alternative multiple-instance learning methods that are based on the notion of finding a “concept” may be used for this purpose if needed. The advantage of identifying a “concept” is that it becomes then possible to visually map the “concept” or its instances back to the corresponding regions in the image. This could be a basis for future work.

The results obtained for the HPA dataset in this paper are meant to illustrate the potential of our approach in feature extraction and grading and its prospect for further extended studies over large datasets and possible combination with complementary approaches that address other aspects of grading (such as nuclear pleomorphism and mitotic count), possibly leading to applications in the clinical context. A comprehensive automated system that would be able to eventually assign high-level grading akin to that by pathologists would undoubtedly have to incorporate, in addition to the work described in this paper, methods that are designed to address nuclear pleomorphism and mitotic count (as most recently in [12]). The final aim is to aid pathologists in the malignancy grading of cancer. As a first step towards that goal, we have in this paper addressed tubule-based grading, which contributes to one of the three components for malignancy grading under the Ellis-Elston system and which is also considered an important factor in Gleason grading.

## Figures and Tables

**Table 1:** Summary statistics concerning the dataset used in this paper.

Dataset	Number of instances	Dimensionality	Number of bags	Number of instances per bag		
				Minimum	Median	Maximum
Breast	1309	40	104	1	10	37

**Table 2:** Classification rates for the breast dataset using different classifiers. Classification was done using 10-fold cross-validation and results are reported as percentage of correct classification ± standard deviation.

Classifier	Classification rate
	Proximity features (Figure 10)
SVC	94.2 ± 2.0
KNN	93.4 ± 0.8
Logistic	80.5 ± 1.3
LDC	61.7 ± 1.2

## References

[1] Elston C. W., Ellis I. O. (1991). Pathological prognostic factors in breast cancer. I. The value of histological grade in breast cancer: experience from a large study with long-term follow-up. *Histopathology*.

[2] Epstein J. I., Allsbrook W. C., Amin M. B. (2005). The 2005 International Society of Urological Pathology (ISUP) consensus conference on Gleason grading of prostatic carcinoma. *The American Journal of Surgical Pathology*.

[3] Prewitt J., Wu S. An application of pattern recognition to epithelial tissues.

[4] Tabesh A., Teverovskiy M., Pang H.-Y. (2007). Multifeature prostate cancer diagnosis and gleason grading of histological images. *IEEE Transactions on Medical Imaging*.

[5] Tabesh A., Kumar V. P., Pang H.-Y. Automated prostate cancer diagnosis and Gleason grading of tissue microarrays.

[6] Doyle S., Agner S., Madabhushi A., Feldman M. D., Tomaszewski J. E. Automated grading of breast cancer histopathology using spectral clustering with textural and architectural image features.

[7] Naik S., Doyle S., Agner S., Madabhushi A., Feldman M., Tomaszewski J. Automated gland and nuclei segmentation for grading of prostate and breast cancer histopathology.

[8] Duin R. P. W. (2011). Non-euclidean problems in pattern recognition related to human expert knowledge. *Proceedings of the International Conference on Enterprise Information Systems (ICEIS '10), Funchal-Madeira, Portugal, June 2010*.

[9] Theodoridis S., Koutroumbas K. (2009). *Pattern Recognition*.

[10] Nowak E., Jurie F., Triggs B. Sampling strategies for bag-of-features image classification.

[11] Zhang J., Marszałek M., Lazebnik S., Schmid C. (2007). Local features and kernels for classification of texture and object categories: a comprehensive study. *International Journal of Computer Vision*.

[12] Mitos-Atypia-14 Mitos & Atypia: detection of mitosis and evaluation of nuclear atypia score in breast cancer histological images. http://mitos-atypia-14.grand-challenge.org/.

[13] Azar J. C., Simonsson M., Bengtsson E., Hast A. (2014). Image segmentation and identification of paired antibodies in breast tissue. *Computational and Mathematical Methods in Medicine*.

[14] Gavrilovic M., Azar J. C., Lindblad J. (2013). Blind color decomposition of histological images. *IEEE Transactions on Medical Imaging*.

[15] Tax D. M. J., Loog M., Duin R. P. W., Cheplygina V., Lee W.-J. (2011). Bag dissimilarities for multiple instance learning. *Similarity-Based Pattern Recognition*.

[16] HPA (2013). *The Human Protein Atlas*.

[17] Dietterich T. G., Lathrop R. H., Lozano-Pérez T. (1997). Solving the multiple instance problem with axis-parallel rectangles. *Artificial Intelligence*.

[18] van Vliet L. J., Verbeek P. W. Curvature and bending energy in digitized 2d and 3d images.

[19] Tax D. M. J. MIL, A Matlab Toolbox for Multiple Instance Learning, version 0.8.1. http://prlab.tudelft.nl/david-tax/mil.html.

[20] Duin R. P. W., Juszczak P., Paclík P., Pękalska E., DeRidder D., Tax D. M. J. A Matlab Toolbox for Pattern Recognition, PRTools4 version 4.2.5. http://www.37steps.com/.

[21] Kuhn H. W. (1955). The Hungarian method for the assignment problem. *Naval Research Logistics Quarterly*.

